# Raising Ecological Awareness and Digital Literacy in Primary School Children through Gamification

**DOI:** 10.3390/ijerph19031149

**Published:** 2022-01-20

**Authors:** María-Carmen Ricoy, Cristina Sánchez-Martínez

**Affiliations:** Department of Didactics, Faculty of Education and Social Work, School Organization and Research Methods, Universidade de Vigo, 32004 Ourense, Spain; c.sanchez@uvigo.es

**Keywords:** educational intervention, environmental education, gamification, ICT, healthy habits, behavior changes, school and family

## Abstract

Environmental education, at least in northwest Spain, is often overlooked in the education system from infant schooling onwards and interventions are needed to raise the profile of this subject. The aim of this study was to examine the impact of a learning program designed for primary school students to broaden their ecological awareness and improve digital literacy using gamification tools. The research was developed using a qualitative approach, with data obtained from 156 subjects, including teachers, students and families. The results show that the children assimilated new habits on the better usage of water and electricity and recycling paper and plastic. Moreover, they acquired more efficient strategies for finding information online, by using apps and developing content with digital tools. Gaming dynamics and resources were the key to students’ learning, with the tablet proving an essential tool for boosting motivation, interaction and problem solving.

## 1. Introduction

Environmental degradation is such a worldwide reality these days that it is humanity’s duty to future generations to tackle this crisis head-on. The greatest challenge is to substantially raise civic awareness of the problem, through reflection and analysis of the original causes of the damage imposed on our planet and its projection onto the environment [1]. Any new initiative must encourage sustainable development and provide innovative solutions to complex problems that take into account economic, socio-educational and environmental factors [2].

The problems of the environment have not been studied sufficiently to provide effective long-term solutions, while the various political and economic initiatives being developed seek to reverse the damage already inflicted on the climate. Another important defense of the environment is to inculcate today’s children with environmental awareness, based on school and family. More research is needed on developing pro-environment behaviors than can be adopted in schools [3]. There are strategies that generally aim to include climate change in educational study plans, yet sustainable development remains an area that needs to be improved [4]. In addition, there is the change caused by scientific communication, and the transition from traditional education to innovative learning supported in media including ICT and gaming. All these changes must be considered in order to enhance scientific communication and the practical implications for education [5,6].

Raising civic awareness requires implementing socio-educational interventions from childhood. Schools can awaken a sense of environmental protection in students through fun activities with the help of families, as part of their overall environmental education. Environmental education, or Education for Sustainable Development (ESD), should be the foundation for building a sustainable future [7]. The United Nations Educational, Scientific and Cultural Organization (UNESCO) [8] identified five areas for immediate action. Firstly, advancing policy; secondly, transforming learning environments; thirdly, developing teachers’ skills and capacities in environmental education; fourthly, suggesting similar initiatives for boosting competencies in students; fifthly, emphasizing the need for action by the community. The United Nations (UN) [9] also put forward an action plan in the form of goals set out in the 2030 Agenda for Sustainable Development, in which it promoted the use of Information and Communication Technologies (ICT) to support ESD, as a means of achieving these objectives.

The aim of this study was to analyze the impact on primary school students’ learning in northwest Spain and on their daily habits in relation to a program to boost ecological awareness and digital literacy through gamification. The design and implementation of this study was based on a diagnostic evaluation that emphasized the need to improve ecological awareness and digital literacy in third-year primary education students; this need has also been detected in other research examined by this study.

The research questions, which represent the characterization/categorization for assessing the intervention developed, were:

- RQ1. Which academic content do the children acquire through educational intervention?
- RQ2. Which ecological habits do the children assimilate through the intervention?
- RQ3. How are ICT and gamification used in children’s learning?
- RQ4. What impact did the intervention have on the children?

The quality of environmental education across all nations needs to improve substantially [10], and the observations of this study can contribute to understanding the importance of students internalizing the academic content, ecological habits and healthy behaviors imparted in environmental education supported by ICT and gamification. We also hope that this study is useful for orienting and stimulating other teachers to design and/or implement their own programs in similar learning contexts.

## 2. Environmental Education, Digital Literacy and Gamification

This has led to increasing interest in environmental sustainability [11]. Overcoming the complex challenges to meeting sustainability goals requires the collaboration and involvement of all of society, not least in education [12].

The concept of sustainable development was first set out in the Brundtland Commission Report of 1987, as a form of development that satisfies the needs of the present without compromising the capacity of future generations to satisfy their own needs [4]. The notion of sustainability is now visible across many different areas when emphasizing human needs; yet there are few studies on the role of education and its efficacy in supporting sustainable development [13]. Advances in this field are still fitful in spite of society’s increasing awareness of the notion. Sustainability and sustainable development need to spread in order for us to continue using today’s resources without undermining quality of life and the well-being of future generations [14].

Environmental education is one element that can help us achieve the UN’s sustainable development goals by 2030. Environmental education in schools should begin early in order to create healthy and environmentally conscious habits in children and awareness of sustainable global development [15]. Educational interventions aimed at citizens are also needed, in order to develop awareness and skills that help people to acquire greater understanding of environmental protection and to promote sustainability [16].

Education policymakers need to rethink the learning process in infant schooling in order to improve students’ understanding of the consequences of environmental degradation for humanity [17,18]. Primary school education is exactly the right time to boost children’s development of responsible behaviors towards protection of the environment [19]. However, there are several barriers to overcome in order for students to internalize the concepts of sustainability, such as the lack of awareness or finance for supporting sustainable actions [20,21].

The Green Schools Foundation in the Republic of Ireland designed an environmental education program called Eco-Schools that has been adopted in 62 countries, reaching more than 16 million students [22]. This initiative addresses a wide range of environmental issues, such as recycling of waste, energy, water and transport. Promoting recycling among primary school students is also important for advancing future sustainable development, and can be actively promoted by educational centers of interest through the 4Rs (reduce, reuse, recycle and recover) campaigns.

Technology can also be applied in the classroom to propose alternative and innovative solutions. An interdisciplinary approach in education that combines theory and practice can foment critical thinking through the use of ICT [23]. Yet, for this to happen, there needs to be a substantial change in current pedagogical strategies; it is here that gamification could make an interesting contribution to innovation in education.

Today’s situation, and the potential of ICT in education, means that boosting digital literacy in schools is more urgently needed than ever. Students’ socio-economic status, school type, digital infrastructure and teachers’ confidence in their own digital skills are all determining factors in the ability to increase digital literacy [24]. So, it suits all interests for a transversal and generalized integration of digital literacy in school study plans; small-scale projects to promote digitalization at school are insufficient [25]. However, implementing specific actions can provide stimulus and a starting point for the development of digital skills and routines that help raise young students’ awareness of caring for their environment. It is evident that many children across the world are already digital natives with permanent access to mobile devices [26].

In general ICT, and mobile devices such as the tablet, enable young people to engage in a vast range of activities that develop new practices and their digital literacy through games, reading, listening and managing content, etc. [27]. Through tablets, students experience activities in a more fun and attractive way, and stimulate competence development in a healthy technological environment [28]. Intuitive and dynamic designs, devices, programs and online apps all offer a range of formats for content production at school [29]. The huge potential of technological devices makes them an appropriate resource for educational programs, with the success of such innovative programs among infants potentially greater if designed around gamification.

Gamification has become increasingly valuable as a methodological resource in primary education, mainly associated with digital technology. Educational strategies can use game design elements in non-game contexts [30] supported in digital resources that involve the student in the learning process. Gamification encompasses three elements: mechanics, dynamics and components. Mechanics refers to aspects that support the game (design, engine and function: script, rules, etc.); dynamics relates to the elements that enable the mechanics to operate, guide and make the process dynamic as the game is played (producing emotions, feedback between people, cooperation, challenges, competition, etc.); components refer to what the gamer wins by exercising their gaming skills (points, levels, insignia, classification or gifts, prizes, etc.) [31].

Gamification as an educational strategy is not effective in itself unless combined with resources and elements that can yield good results [32]. The use of gamification strategies in educational contexts should enable children to enjoy gratifying experiences that relate to their everyday lives. The main aim of these strategies is to develop problem-solving skills and improve their daily habits in order to achieve effective change in behaviors [33].

Digital devices, such as the tablet supported in gamification, can actively engage the student [34]. Learning environments that promote autonomous learning and interaction are a valuable resource for training students [35,36], while digital mobile devices and gamification in learning are now recognized as tools with enormous potential in infant education [37].

## 3. Context, Design and Implementation of the Intervention

The intervention was aimed at third-year primary school students aged 8–9 at three education centers in northwest Spain. As in most countries, primary education is free and compulsory in Spain. Primary school education covers six academic years of study for students aged 6–12. ICT are integrated in primary school study plans, with primary education’s objectives including the development of basic technological competences and ICT use in learning, and the development of critical thinking [38].

The choice of the three school centers for the study rested on access provided by the management at the centers that agreed to implement the program. These centers had performed a diagnostic evaluation to detect student needs in terms of their awareness of protecting the environment. The diagnostic evaluation was based on information gathered from primary school students in four discussion groups. The questions and dialog used focused on their academic learning about protecting the environment and the digital resources they used. An open-question survey sent out to the families of these students also provided information on their children’s ecological habits. The information supplied to the researchers showed that the students’ only exposure to the study topic was in Natural Sciences and Social Sciences classes, with a traditional activity format; use of digital resources for the subject was scant. However, the diagnosis showed that the students enjoyed using the tablets they had at home, with the support of their parents. The school centers selected were viable for implementing the educational intervention as they had good technological resources and their students were of sufficient maturity to engage with the subject.

Based on the exploratory study and other studies developed by different authors [10,15,39], we designed an educational program whose aim was to raise environmental awareness in primary school children to take care of the environment, and improve their digital literacy skills through gamification. The program adopted a transversal approach around the 4Rs (reduce, reuse, recycle and recover) campaigns. Our ecological program was designed and implemented by external teachers in the final term of the school year; the program was used with four groups of students in eight sessions that took place over four weeks. Each group had two sessions per week of 50 minutes’ duration (Table 1). 

The intervention was sequenced in order to assess how the students were learning during the program rollout. Students’ immersion in the program’s activities was gradual and determined by strategies supported in brainstorming and debate. Continuous evaluation was monitored by the e-diaries kept by students and the teacher who implemented the program. In the final evaluation, a table was used to register the level of success, which enabled us to extract the results from the praxis developed with the children.

## 4. Materials and Methods

The research was qualitative, and developed from a broad case study. The qualitative methodology allowed us to describe and understand the reality analyzed, and perform an in-depth analysis of the peculiarities of the study context [40], taken to be an organizational set [41]. The variety of cases gave us a huge trawl of data that bolstered the solidity and validity of the results [42].

### 4.1. Participants, Instruments and Data Gathering

The study took place in three educational centers in northwest Spain, involving 156 subjects. There were 83 students spread across four groups: Center 1 (25 children in the third year of primary school, Group A—13 boys, 12 girls; 25 children in the third year, Group B—13 boys, 12 girls); Center 2 (24 children—9 boys, 15 girls); Center 3 (9 children, —7 boys, 2 girls). Families also participated (14 fathers, 54 mothers). There were five teachers (4 women, 1 man) aged 28–47 (=36 years), with teaching experience ranging from 1 to 20 years.

**Figure 1:**
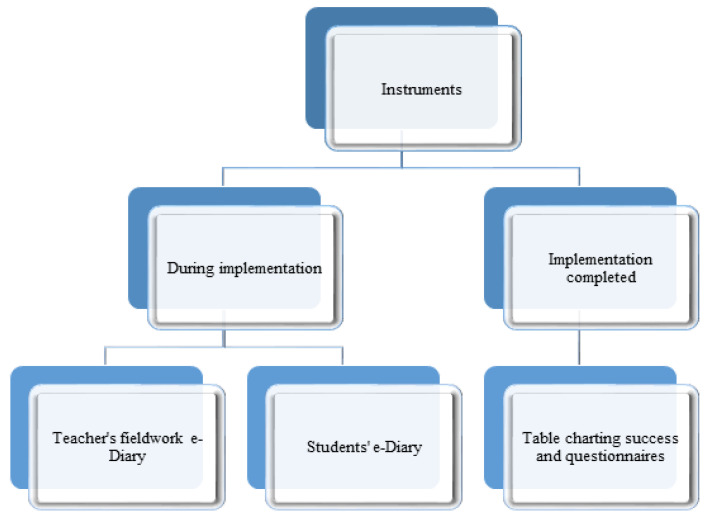
Data-gathering instruments.

Various instruments were used to gather data on the intervention (Figure 1): a teacher’s fieldwork e-diary, students’ e-diaries, a table charting success and questionnaires. The instruments were developed ad hoc as none were available in the market that could be adapted to this research. They were constructed following the common procedure for qualitative research to guarantee validity and consistency. The instruments were first designed by two researchers and authors of this work, and then tested by three experts in the study topic and in the techniques developed, which guaranteed the aptness and pertinence of the tools designed.

The various instruments used in this research facilitated the gathering of the narrative data, the most widely used in qualitative research [43]. The instruments were adapted according to the type of collective and information gathered.

The e-diaries of the children and external teacher were used as a data source. The e-diary is a useful instrument for the analysis, description and evaluation of everyday school life [44]. The aim of the children’s e-diaries was to systematically collate the development of their first-person experiences.

The children used their e-diaries to reflect on the ecological habits they were acquiring and on the changes assimilated in the activities that were developed. They completed their e-diaries at the end of each session. The external teacher who implemented the program systematically monitored the evolution of the children during the program, the use of ICT resources and the contribution of gamification. These instruments are the same as those used in the continuous evaluation of students during the academic year.

In the final evaluation, the teacher logged the level of success assigned to each student. This was achieved with a table that charted success, registering aspects observed in each student and their evolution. The table was structured in columns to facilitate the data gathering. Another instrument was the open-question survey, commonly used in education as an easy and appropriate way to gather narrative data [45]. The final phase also included open-question surveys in order to know the extent of the learning acquired by the students, and to show the changes of habit or behavior identified by the teacher and the families involved. 

### 4.2. Data Analysis

A content analysis was performed on the data collected by coding and categorizing the data patterns obtained. The categorization was initially debated among a group of five experts who supported the researchers in this study. Five working sessions were needed to identify the units of meaning that were the object of this analysis. The analysis categories were constructed around the nexus of the study objective and the research questions (Table 2). 

**Figure 2:**
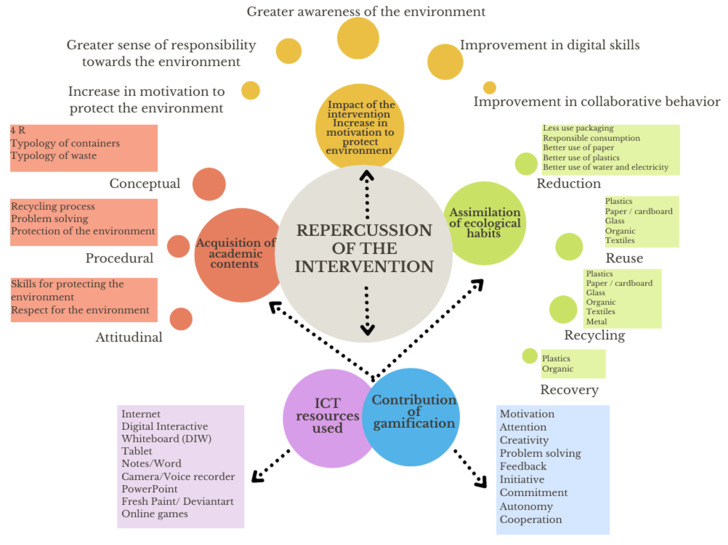
Hierarchy of categories.

This categorization resulted from a deductive and intuitive process (of the existing theory) based on a naturalist conception that emerges in the data gathered [47]. AQUAD version 7 (Tübingen, Baden-Wurtemberg, Germany) and Excel were used in the content analysis. The technology helped to strengthen the development of the procedure followed in the analysis which, in turn, was reinforced by expert guidance. The hierarchy of categories presents an itemization of the different levels of categories obtained together with their definitions (Figure 2).

## 5. Results

For analysis, the results of this study were grouped in two subsections, to match the study objectives and research questions.

### 5.1. The Children’s Acquisition of Academic Content (RQ1 and RQ3)

The teachers stated that the students acquired some of the conceptual, procedural and attitudinal content relating to the protection of the environment and ICT (Table 3). They all agreed (4/4; 100%) that the best assimilated concept was the 4Rs (reduce, reuse, recycle and recover). One teacher stated that the children understood the concepts of the classification of waste containers used to distribute rubbish (1/4; 25%); another teacher said students had assimilated the concepts associated with waste (1/4; 25%).

**Figure 3:**
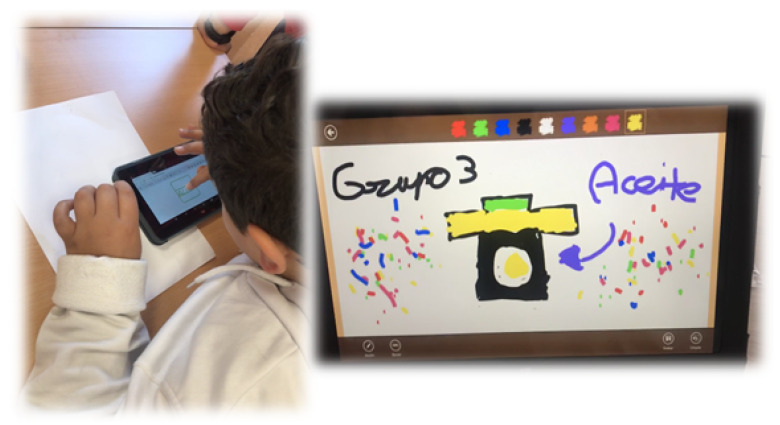
A child’s drawing of a container for collecting used cooking oil.

The data showed that ICT were essential tools for student learning on the environment. The most prominent digital resources used in the intervention were the digital interactive whiteboard (DIW), the tablet and its various apps (notes, camera, voice recorder, etc.), as well as other external apps for drawing and online interactive games, and the Internet. For example, the children designed a container for collecting and recovering used cooking oil (Figure 3) using the Fresh Paint and Deviantart apps.

The use of ICT supported in gamification contributed to increased motivation for learning, and improved attention and concentration in class. Moreover, the teachers stated that the technology and gamification stimulated the children’s creativity and the development of cooperation and problem solving, which reinforces autonomous learning. This underlines the increasing interest in the use of methodological resources backed by gamification to stimulate interaction, cooperation, imagination, autonomy, etc., as one example from the survey indicates:

*I consider that the implementation of the program has been a success and that the children have learnt a lot. The fact that they used the tablet and could learn using different play resources was fun for them and motivated them a lot. They learnt new theoretical aspects and also become more ecologically aware. They shared their new habits and attitudes on recycling with their friends and family, and this is how we all win!*

*(Teacher, aged 28, school SC. Lines of analysis 10–14).*

Half the teachers involved in the study (2/4; 50%) stated that the children had internalized procedures on protecting the environment. The teachers were surprised by how much the children now saved in electricity and water, or adhered to guidelines (to reduce consumption of energy, waste and paper) that the children themselves had designed on card or digitalized with the tablet camera. The teachers saw how the children improved their capacity for problem resolution (as in the production of a video to raise people’s awareness of caring for the environment), and how they assimilated the process of recycling, by separating waste and depositing it in the appropriate container (1/4; 25%). The teachers also commented on how the children had internalized new attitudes on respect for the environment (3/4; 75%) and absorbed skills and habits for caring for their surroundings (1/4; 25%). For example, they became more motivated to water and protect plants.

**Figure 4:**
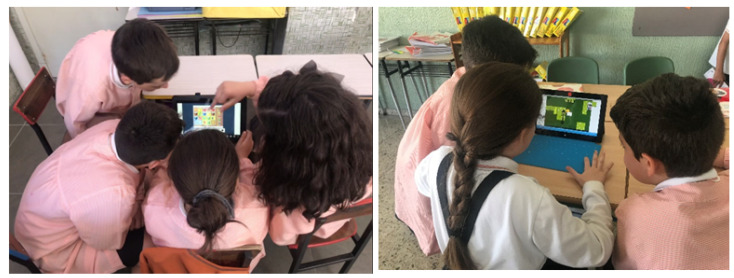
Children playing the online game “Los Cokitos”.

The acquisition of procedural and attitudinal content was mainly supported in technological resources (DIW, tablet, Internet). Apps on the tablet (voice recorder, camera for photos and video) were used to good effect, along with others on the Internet, such as games. The teachers emphasized the children’s interest in new apps for interactive games on recycling (Figure 4), such as Los Cokitos (https://acortar.link/12snu9) (accessed on 17 June 2021) and Pocoyo (https://acortar.link/9VcFL6) (accessed on 17 June 2021). These online games require the student to classify different types of waste (plastic and cans, paper, glass and organic material) and deposit them in the appropriate container (yellow, blue, green igloo-shaped or brown). The children’s acquisition of knowledge and skills was boosted by the use of gamification strategies, through infant-level scripts/texts, game rules, incentives to develop emotions, feedback and dynamics, all of which encouraged student participation. Such elements stimulated the children to boost their scores and ranking based on the work they developed. All these factors helped to motivate the students to protect the environment, aroused their curiosity and stimulated individual initiative and cooperation.

### 5.2. Assimilation of Ecological Habits and Impact of the Intervention (RQ2, RQ3, RQ4)

The children’s families and teachers believed that the students had internalized valuable habits and behaviors for protecting the environment based on waste recycling (Table 4). For example, they saw how the children had reduced their use of plastics (48/83, 57.83%; 2/4, 50%) and paper, by using a single sheet of paper for different tasks (68/83, 81.93%; 8/33, 24.24%; 1/4, 25%), and had made much more ecological use of other natural resources (71/83, 85.54%; 8/33, 24.24%) such as sunlight and water.

The gamification strategies were mainly supported in ICT. The Notes/Word app and presentation programs such as PowerPoint were used to present certain guidelines for promoting the reduction of waste material. First, the teacher encouraged students to form small groups to use Notes/Word to draw up some rules, then they designed representations of these rules using PowerPoint; after that, they printed and placed them on a classroom mural. The children enjoyed performing these activities, allowing them to develop their imagination and acquire new knowledge. The following is an extract from a parent:

*The intervention has been really important for my daughter. For example, she has reduced the amount of water she uses (using less water in the bathroom) and electricity (she now only turns on the light when absolutely necessary). She really enjoyed the activities in the program (she loved using the tablet and the apps). Her daily behavior regarding the environment has improved and she is much more aware of protecting the environment.*

*(Father, aged 43, School F. Lines of analysis 12–16)*

Following the intervention, the children reused plastic both at home and in the classroom (72/83, 86.75; 12/33, 36.36%; 2/4, 50%). For example, they used plastic water bottles in different ways, refilling them with tap water or from a bigger water bottle for several days; glass bottles (36/83, 43.37%; 7/33, 21.21%; 1/4, 25%) and paper and cardboard (63/83, 75.90%; 6/33, 18.18%; 1/4, 25%) were also reused. The children recovered notebooks from previous academic years that still had blank pages; they also used cardboard packaging from everyday products, such as cartons, for manual crafts classes (to make pencil holders, organizers etc.).

The children’s acquisition of new habits was stimulated largely by the use of apps such as Google and YouTube, supported by DIW and the tablet. For example, children would first watch a video, which in turn stimulated them to generate creative ideas for making plant pots from reusable materials. Producing these plant pots represented a challenge for the students, in which they experienced a range of emotions and considerable satisfaction on completing the task. This sense of achievement increased their capacity to solve problems and boosted their creativity, autonomy and motivation for learning.

As a result of the intervention, the children actively separated waste into the appropriate container: glass (46/83, 55.42%; 12/33, 36.36%; 1/4, 25%), plastics (79/83, 95.18%; 11/33, 33.33%; 3/4, 75%) and paper or cardboard (67/83, 80.72%; 10/33, 30.30%; 4/4, 100%). The teachers (3/4, 75%) and children (67/83, 80.72%) agreed that they were now more aware of recycling organic material. For instance, they separated banana skins and orange peel and placed them in the organic material bin at home. The assimilation of ecological habits was generated by a focus on recycling, encouraging the children in a playful format to produce adverts on the subject (using the tablet voice recorder and camera). The students also took part in online games on recycling. The dynamics of these games presented them with a challenge, to score as many points as possible by collecting/depositing waste in the correct container within a certain time limit, and to win prizes based on correctly answering questions. The latter function was a good motivator for their learning, as the following extract shows:

*In general, the program helped improve the children’s learning. I believe that the children have become more aware of protecting the environment and recycling. The program introduced them to the 4Rs, procedures related to recycling and attitudes of respect for the environment. Now, the children use less paper and reuse old notebooks that still had unused pages, and recycle material that we consume in the classroom: plastic bottles, paper and cardboard, organic material such as food leftovers from break time (depositing each item in the corresponding waste bin). I think that using the table and gamification strategies has increased their motivation for learning. Experimenting with online games has also encouraged them to work in collaboration with each other.*

*(Teacher, aged 35, school FA. Lines of analysis 15–21).*

Waste recovery actions were the least familiar to both students and their families; they only recovered plastics (2/33, 6.06%) and organic material (36/83, 43.37%; 4/33, 12.12%; 2/4, 50%). According to the parents, the recovery of plastics consisted of gathering plastic bottles and bottle tops, small yogurt tubs, etc. In the intervention, the children participated in an action to recover used cooking oil, depositing the liquid in its corresponding container (first phase of the recovery process). Other activities to promote recovery actions were developed using drawing apps, in which the children were explained the rules then worked in groups to design an orange container (symbolizing the collection point for used cooking oil). This activity involved different gaming strategies to foment critical thinking, creativity and cooperation.

Overall, the impact of the intervention proved very positive for improving children’s learning and internalizing of ecological habits (Table 5). After the program, the children’s level of awareness of the importance of caring for the environment and of the consequences of its degradation increased. These changes were apparent in the new ecological habits the children now adopted at school and home (level of success “excellent”: 80/83, 96.39%).

**Figure 5:**
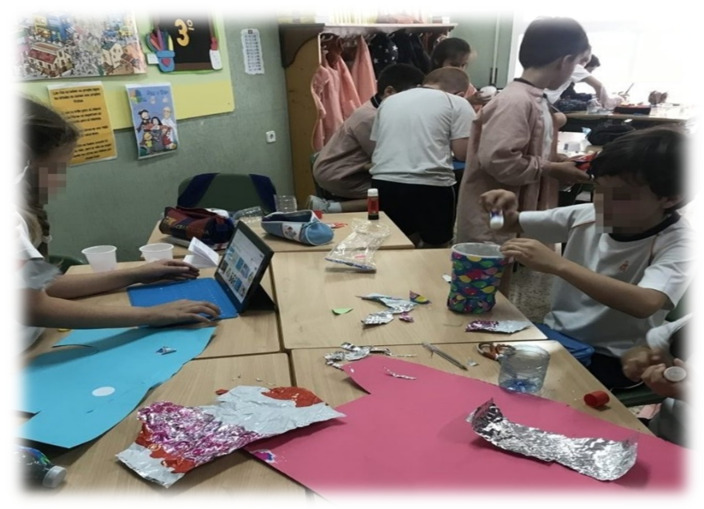
Students working in a group with a range of materials.

The children’s use of ICT during the intervention boosted their digital literacy process (level of success “excellent”: 76/83; 81.57%), mainly developed using fun activities. These included searching the Internet to locate audiovisual material, recording audio (to compose a children’s song that expressed concern about the environment) or video (to produce a video to raise people’s awareness of environmental protection). The program involved a greater amount of time using ICT, under the guidance and supervision of the teacher and family, which encouraged a healthy use of digital resources by the students. In general, ICT use supported in gamification contributed substantially to the children’s involvement in protecting the environment (level of success “good”: 41/83; 49.40%). The students raised their level of environmental responsibility by assuming guidelines and actions on protecting the environment, both at school and at home (level of success “excellent”: 72/83; 86.75%). These activities were aimed at reduction, reuse, recycling and recovery of waste. Some children greatly improved their collaborative skills (level of success “excellent”: 32/83; 38.55%), while the majority reached a “good” level (44/83; 53.01%) (Figure 5).

## 6. Discussion

Much environmental degradation is caused by humankind, but our damaging actions can be eradicated and our good actions improved by providing appropriate learning and raising awareness in children from an early age. Civic awareness of the gravity of the problems facing the environment has generally increased in the last decade, but there still exist erroneous concepts governing the actions that individuals can take to safeguard the environment [48]. The potential of technological and innovative solutions generates the confidence to drive forward the motivational and behavioral processes that develop positive attitudes towards the environment [49]. With environmental education at the initial stages of learning, innovative educational programs can help to raise students’ ecological awareness. A recent study has stated that games are a good way to inform, motivate and alter the attitudes and behaviors of students as they confront the problems and challenges of sustainability [50].

The ecological program implemented, mainly supported in technological and gamification resources, enabled students to assimilate relevant curricular content at school, with the support of family (mothers and fathers). Other research has demonstrated an increase in students’ knowledge of sustainability following the use of innovative programs on this subject [51]. The children acquired content on the protection of the environment and recycling based on the 4R activities; for a better future for all, it is vital that young students learn scientific concepts and recognize the value of promoting sustainability [21]. Recycling has become a reasonably well-developed civic habit but the economic capital and energy needed to transport and transform waste into recyclable material has declined worldwide. Thus, effective practices are still needed to boost the reduction and reuse of waste as alternatives to merely recycling [52].

The newly acquired ecological habits learnt by the children during the intervention continued afterwards with a reduction in consumption of plastics and energy, both at school and in the home. The program enabled students to internalize routines to reuse plastics and glass. In other contexts, the “4energy” initiative (in Spanish state schools) has raised students’ awareness of saving energy by instructing them on responsible behaviors in this area [53]. Unfortunately, worldwide production of plastic in 2019 reached 370 million tons, of which only 9% had been recycled and 12% incinerated, with the rest dumped in the environment or in landfills. Plastic littering of the land and sea is currently at an all-time high, making it essential that our children develop good environmental habits to reduce, reuse, recycle and recover plastic [54]. Children can progressively acquire the habits of recycling glass, paper and cardboard, or learn to recover organic waste such as used cooking oil, all of which can be imparted using fun activities at school. For example, schools and families can involve children in producing soap from oil, making minor house repairs such as oiling door hinges or locks, helping to repair furniture, etc.

This study has shown that the impact of the intervention on students’ learning was very positive, helping to raise awareness of protecting the environment. The children also improved their digital skills, for example, learning how to search for specific information on the Internet, and by working with drawing apps and interactive games. It is clear that a greater use of ICT in education improves children’s digital competence in the short term and can instill positive behaviors. Other research has found that a high degree of environmental literacy and awareness generates a better grasp of scientific vocabulary that can manifest in more efficient and assertive communication when people (according to age and characteristics) become involved in socio-environmental issues [55].

Achieving the goal of sustainable development is ambitious for any economy, society and the environment [56]. For this reason, citizens must start to acquire awareness and knowledge of the environment from an early age in order to adapt their behavior, daily routines and attitudes. Preserving our immediate physical environment today is essential, and this requires encouraging children to participate in activities that contribute to successful sustainable development [57]. Such activities will predictably have a beneficial effect on the quality of life of children and on the environment.

This intervention was mainly supported in ICT resources such as DIW and mobile devices including tablets, and the Internet. A range of computer programs and videogames were used, with the latter particularly motivational and fundamental for raising students’ awareness of protecting the environment and, as in the case of other interventions, contributing to fomenting new attitudes and behaviors that respect the environment [58]. Likewise, other non-digital materials proved equally useful in the intervention, including waste products (plastic bottles, glass jars, paper, cardboard, etc.) and stationery (card, scissors, glue, pens, etc.).

The intervention showed that the main contribution of gamification to children’s learning was the increase in motivation, creativity, problem-solving skills and cooperation. The use of game mechanisms, dynamics and components stimulated the children to undertake actions and boosted their learning [30]. Active methodologies, such as learning based on problem resolution or gamification, helped students solve the problems posed [59]. Other studies have also shown that gamification in education incentivizes students to learn and increases their participation, interaction and commitment [60].

Gamification has huge potential for improving digital competence, as numerous high-impact studies have shown in recent years [61]. The use of fun strategies to help children develop their learning (with a premium on action, experimentation, conflict resolution and interaction between students, etc.) at school and at home, supported by ICT resources, increases the positive impact of a range of everyday skills on the environment and on the development of academic knowledge.

In summary, it should be noted as the main achievements of the implementation of the program, the children have:

- Acquired new content related to the protection of the environment and recycling, based on actions associated with the 4Rs;
- Internalized some valuable environmental habits or behaviors, mainly through waste recycling;
- Made more used of ICT resources such as DIW, mobile devices (such as the tablet) and the Internet. They have used new digital applications and different video games. Gamification was especially stimulating for raising awareness among pupils about environmental protection, and has contributed to generating new collaborative or respectful attitudes and problem-solving skills;
- Improved the learning and internalization of some ecological routines. In addition, children have strengthened their digital literacy process with the gamification processes used, with the environment as the focus of study.

Finally, it should be noted that in order to monitor the program [62], it is important to continue generating and strengthening ecological habits in children, through digital gamification, and to know its impact in the medium term. The mechanisms for carrying out the monitoring study of the program can be diverse, depending on the context in which it has been implemented. However, some general guidelines can help to make decisions on how to guide the educational intervention process [63,64]. Among others, the following may be recommended: Group assessment, based on discussions on the explanations/arguments that children have in relation to the preservation of the environment; Recording the degree of satisfaction and/or involvement that children manifest/show with the development of daily actions they carry out to preserve the environment; Systematic observation and analysis of the main obstacles that influence the consolidation of the new habits of students associated with the protection of the environment; Analysis of the schoolwork carried out by students on the 4Rs (to ascertain the consolidation and increase of theoretical content, the development of pro-environmental attitudes, sensitivity, beliefs, etc.), with appropriate feedback from teachers.

## 7. Conclusions

The school and the family are excellent contexts for children to learn and improve habits and behaviors, and internalize guidelines for the protection of the environment. Actions that incorporate the 4Rs (reduce, reuse, recycle and recover) are ideal for working with children from an early age.

Following the intervention on raising awareness about protecting the environment, students could better understand and assimilate ecological routines and behaviors based on recycling, both at school and home. As a result, the children consume less paper and reuse more plastic, recycle glass and plastics and recover waste products. The use of digital tools supported in gaming strategies is an excellent medium for motivating children to take more care of the environment, and to awaken and reinforce their environmental awareness.

## 8. Limitations and Future Research Lines

This investigation was based on the participation of students, teachers and parents, but it would be interesting to broaden participation to develop a quantitative study. Another limitation of the sample is that it was restricted to a single academic year (third-year primary school students). Although three different schools were involved, the intervention could be extended to include more school centers, other educational phases and regions. It would also be useful to have the design of this program assessed in order to optimize it for future interventions, and also extend the implementation time. The future potential of this research can be discussed in terms of being a qualitative and small-scale study, and for possible bias, so, it would be interesting to carry out a study on the longevity of the impact on children of the effect of their ecological behavior.

## Figures and Tables

**Table 1:** Objectives and activities of the educational program.

N° Session	Didactic Objective	Title and Brief Description
1	• To understand the consequences of polluting the environment, especially in the students’ own environment. • To develop communication skills in relation to the environment.	Do we know the situation of own environment today? Didactic audiovisual material was shown to students on the problems facing the environment, followed by reflection and analysis through brainstorming and discussion; composition and recording of a children’s song to express their vision of environmental problems.
2	• To internalize norms that encourage reduction of consumption of energy, waste and paper. • To develop habits for respecting the environment; develop skills for online searching and filtering of information on the environment.	First stop: VillaReduce: a presentation was developed with the children using digital programs and apps to focus on guidelines for reducing consumption of energy, waste and paper.
3	• To explore mechanisms for the reduction of consumption of energy, waste and paper.	Let’s cover VillaReduce with posters! Poster-making session using colored cardboard to visualize the guidelines discussed in session 2, to promote reduction of consumption of energy, waste and paper. The students then digitalized the posters using the tablet camera.
4	• To develop skills for making objects from reusable materials. • To appreciate how reusing materials enables us to take better care of the environment.	Second stop: VillaReutiliza: The children produced plant pots from reusable materials they had brought from home, with the help of their family.
5	• To develop mechanisms to distinguish the different types of waste and their corresponding containers. • To improve recognition capacity in order to correctly classify/separate waste.	Third stop: VillaRecicla: A short children’s documentary was shown, followed by a debate; videogames were used to examine the question of waste separation.
6	• To understand the importance of recycling in caring for the environment. • To identify aspects of environmental contamination. • To initiate students in the creation of multimedia to promote recycling.	We are all ambassadors of VillaRecicla! The children were encouraged to produce an advert, using the tablet’s camera and voice recorder, to promote recycling.
7	• To recognize the importance of recovering waste in order to take better care of the environment. • To develop skills to identify waste collection locations. • To initiate students in graphic production to promote waste recycling.	Fourth stop: VillaRecupera: The students watched some children’s video shorts on collection and recovery of used cooking oil, followed by the children producing creative designs for used cooking oil collection containers on an app.
8	• To understand the process of seed planting in objects made from reusable materials; to aid environmental protection. • To identify the consequences of environmental contamination.	To end our journey…let’s create our own vegetable garden! The children collaborated to make an ecological vegetable garden by planting seeds in the plant pots produced in session 4.

**Table 2:** Main category definitions.

Categories: 1st Level	Categories: 2nd Level	Category Definition
Acquisition of academic content	Conceptual Procedural Attitudinal	Typology of academic content acquired by the student in the conceptual, procedural and attitudinal aspects [46]
Assimilation of ecological habits	Reduction Reuse Recycling Recovery	The 4Rs (reduce, reuse, recycle and recover) that the student gradually comes to adopt during the intervention.
ICT resources used	Internet Digital Interactive Whiteboard (DIW) Tablet Notes/Word Camera/Voice recorder PowerPoint Fresh Paint/Deviantart Online games	All the different ICT resources used during the implementation.
Contribution of gamification	Motivation Attention Creativity Problem solving Feedback Initiative Commitment Autonomy Cooperation	The contribution of gamification strategies to the intervention.
Impact of the intervention	Increase in motivation to protect environment Greater sense of responsibility towards environment Greater awareness of environment Improvement in digital literacy skills Improvement in collaboration skills	The reach of the intervention in the primary school students.

**Table 3:** Students’ assimilation of the academic content.

Categorization			Teachers/Tutors (*n* = 4)	Teacher Who Implemented the Program																
				ICT Resources Used								Contribution of Gamification								
1st Level	2nd Level	3rd Level	Percentage (%)	Internet	DIW	Tablet	Notes/Word	Camera/Recorder	PowerPoint	Fresh Paint/ Deviantart	Online Games	Motivation	Attention	Creativity	Problem Solving	Feedback	Initiative	Commitment	Autonomy	Cooperation
Acquisition of Content	Conceptual	4R	100	✓	✓	✓	✓	✓	✓	✓	✓	✓	✓							
		Typology of containers	25	✓	✓	✓				✓	✓	✓	✓	✓	✓	✓				✓
		Typology of waste	25	✓	✓	✓	✓			✓	✓	✓	✓	✓	✓					✓
	Procedural	Recycling process	25	✓	✓	✓		✓		✓	✓	✓		✓	✓	✓				✓
		Problem solving (guidelines for reducing consumption)	25	✓	✓	✓	✓	✓	✓		✓	✓		✓	✓		✓		✓	✓
		Protection of the environment (more economical use of water and electricity, complying with guidelines for reduction, etc.)	50	✓	✓	✓				✓	✓	✓		✓	✓	✓	✓	✓	✓	✓
	Attitudinal	Skills for protecting the environment	25	✓	✓	✓	✓	✓	✓		✓	✓		✓	✓		✓	✓	✓	✓
		Respect for the environment (plants, animals, etc.)	75	✓	✓	✓		✓	✓			✓					✓	✓	✓	✓

Note: Light gray refers to ICT Resources Used; Medium gray refers to Contribution of Gamification; Dark gray refers to absence/empty.

**Table 4:** Internalized ecological habits based on 4R activities.

Categorization			Students (*n* = 83)		Families (*n* = 33)		Teachers Tutors (*n* = 4)	Teacher Who Implemented the Program																
								ICT Resources Used								Contribution of Gamification								
1st Level	2nd Level	3rd Level	fi	n_i_	fi	n_i_	n_i_	Internet	PDI	Tablet	Notes/Word	Camera/Recorder	Power Point	Fresh Paint/Deviantart	Online Games	Motivation	Attention	Creativity	Problem Solving	Feedback	Initiative	Commitment	Autonomy	Cooperation
Assimilation of Ecological Habits	Reduction	Less use of packaging	33	39.76	5	15.15		✓	✓	✓	✓		✓			✓	✓	✓	✓		✓	✓	✓	✓
		Responsible consumption	39	46.99	6	18.18	25																	
		Better use of paper	68	81.93	8	24.24	25																	
		Better use of plastics	48	57.83			50																	
		Better use of water and electricity	71	85.54	8	24.24																		
	Reuse	Plastics	72	86.75	12	36.36	50	✓	✓	✓						✓		✓	✓				✓	✓
		Paper/cardboard	63	75.90	6	18.18	25																	
		Glass	36	43.37	7	21.21	25																	
		Organic			1	3.03																		
		Textiles	11	13.25	1	3.03																		
	Recycling	Plastics	79	95.18	11	33.33	75	✓	✓	✓		✓			✓	✓			✓	✓	✓	✓	✓	✓
		Paper/cardboard	67	80.72	10	30.30	100																	
		Glass	46	55.42	12	36.36	25																	
		Organic	52	62.65			75																	
		Textiles	21	25.30	5	15.15																		
		Metal			5	15.15	25																	
	Recovery	Plastics			2	6.06		✓	✓	✓				✓		✓		✓					✓	✓
		Organic	36	43.37	4	12.12	50																	

Note: fi = absolute frequency; ni = relative frequency. Light gray refers to ICT Resources Used; Medium gray refers to Contribution of Gamification; Dark gray refers to absence/empty.

**Table 5:** Impact of the intervention on the children.

Category (1st Level): Reach Generated by the Intervention	Level of Success Achieved by the Students				
Sub-Categories (2nd Level)	Excellent (fi, ni)	Good (fi, ni)	In Process (fi, ni)	Insufficient (fi, ni)	
Increase in motivation to protect the environment	34	41	6	2	Data provider: Teacher. N° of students: 83
	40.96	49.40	7.23	2.41	
Greater sense of responsibility towards the environment	72	9	2	0	
	86.75	10.84	2.41	0.00	
Greater awareness of the environment	80	2	1	0	
	96.39	2.41	1.20	0.00	
Improvement in digital skills	76	5	1	1	
	91.57	6.02	1.20	1.20	
Improvement in collaborative behavior	32	44	6	1	
	38.55	53.01	7.23	1.20	

Key: fi = absolute frequency; ni = relative frequency.

## Data Availability

Not applicable.
